# Enhanced Recovery After Surgery Protocols in Major Urologic Surgery

**DOI:** 10.3389/fmed.2018.00093

**Published:** 2018-04-09

**Authors:** Natalija Vukovic, Ljubomir Dinic

**Affiliations:** ^1^Anesthesiology and Reanimation Center, Clinical Center Nis, Nis, Serbia; ^2^Urology Clinic, Clinical Center Nis, Nis, Serbia

**Keywords:** urology, recovery, radical cystectomy, enhanced recovery after surgery, fast-track pathway

## Abstract

**The purpose of the review:**

The analysis of the components of enhanced recovery after surgery (ERAS) protocols in urologic surgery.

**Recent findings:**

ERAS protocols has been studied for over 20 years in different surgical procedures, mostly in colorectal surgery. The concept of improving patient care and reducing postoperative complications was also applied to major urologic surgery and especially procedure of radical cystectomy. This procedure is technically challenging, due to a major surgical resection and high postoperative complication rate that may reach 65%. Several clinical pathways were introduced to improve perioperative course and reduce the length of hospital stay. These protocols differ from ERAS modalities in other surgeries. The reasons for this are longer operative time, increased risk of perioperative transfusion and infection, and urinary diversion achieved using transposed intestinal segments. Previous studies in this area analyzed the need for mechanical bowel preparation, postoperative nasogastric tube decompression, as well as the duration of urinary drainage. Furthermore, the attention has also been drawn to perioperative fluid optimization, pain management, and bowel function.

**Summary:**

Notwithstanding partial resemblance between the pathways in major urologic surgery and other pelvic surgeries, there are still scarce guidelines for ERAS protocols in urology, which is why further studies should assess the importance of preoperative medical optimization, implementation of thoracic epidural anesthesia and analgesia, and perioperative nutritional management.

## Introduction

The new era in perioperative medicine, defined as enhanced recovery after surgery (ERAS) protocols, started with the increase of the importance of a multimodal approach to surgical patients. The most important aims of the multimodal approach are the improvement of patients’ preoperative status and the perioperative maintenance of homeostasis, by minimizing stress response and inflammation. This new approach was first used in colorectal surgery (1) and then started spreading to all other types of surgeries (2–4).

There is an increased interest in ERAS protocols in urology. Radical cystectomy (RC) and radical prostatectomy (RP) are predominantly studied urologic procedures. These procedures have major surgical resection, increased risk of bleeding and perioperative transfusion, and in case of cystectomy, urinary diversion and high frequency of postoperative complications. Furthermore, patients undergoing major urologic surgery are usually the elderly, with cardiovascular and other comorbidities, anemia, possible infection, and malnutrition.

## Preoperative Period

The ERAS preoperative period consists of several important elements, which are described below.

### Preadmission Counseling and Education

When it comes to ERAS guidelines, the initial phase of different surgeries is preadmission counseling (4–6). It has been determined that the reduction of anxiety by means of sharing details of admission, as well as surgical and anesthetic procedures, may improve pain control (7), early mobilization, and perioperative feeding and hence reduce postoperative complications (8, 9).

Even before first studies of ERAS in urology, Hobisch et al. (10) found out that 65–71% of the patients scheduled for different types of urinary diversion RC had received no information about various therapy options before being admitted to the Department of Urology. After admission, 78.8% of ileal conduit patients and 91.3% of neobladder RC patients were completely satisfied with the information given. Most of the studies from this period (10, 11) mainly examine the need to explain different types of RC urinary diversions to patients and the impact they will have on everyday care after hospitalization. The importance of preadmission counseling and patient education, along with a precise and detailed clarification of the immediate perioperative pathway was not emphasized. To the best of our knowledge, there are no specific studies dealing exclusively with preadmission counseling and education. This issue has so far been analyzed only as a part of the study of ERAS elements.

Dutton et al. (12) examined 165 patients undergoing open RC with urinary diversion implementing ERAS protocol. Patients in this study went through three stages of patient education and counseling. The first stage involved pre-referral by patient’s family doctor, the second one was outpatient assessment by a nurse specialist, and the third one included preoperative patient education (both written and verbal) explaining ERAS, as well as stoma or neobladder care before hospital admission. The authors pointed out that the patients were informed at earliest opportunity. Taking everything into account, it can be concluded that the implementation of ERAS elements in this study was safe, coupled with early feeding of patients, early mobilization, and rapid discharge from hospital.

In the following years, more researchers (13, 14) have included preadmission counseling as an obligatory element of ERAS protocols in urology. In the prospective study of Pang et al. (14), preoperative counseling included detailed 30–40 min description of the treatment, provided by a surgeon, a nurse, a stoma therapist, and an anesthesiologist, if necessary. In this study, the patients were additionally preoperatively provided with an information booklet.

Matulewicz et al. (15) also suggested the use of multimedia tools (websites and videos) and intensive verbal and written counseling regarding the expectations and the goals of RC. The authors furthermore proposed the implementation of “Urostomy Education Scale” (16) as an important tool for patient education in patients with urostomy after RC. Moreover, within the systematic review (17), the importance of RC patients’ participation in advocacy networks was also stressed, with the aim of improving perioperative care, both before and after surgery.

### Preoperative Optimization

Preoperative optimization involves assessment and improvement of medical conditions, as well as the reduction of risks that affect perioperative homeostasis. The guidelines provided by the European Society of Anaesthesiology on preoperative evaluation (18) recommend different strategies that should be used for reducing perioperative risks.

Current or former smokers comprise 80% of RC patients (15). Preoperative smoking cessation might reduce the risk of pneumonia, mechanical ventilation longer than 48 h, and unplanned tracheal intubation. According to the study of Turan et al. (19), active smokers have higher rates of myocardial infarction, postoperative cardiac arrest and stroke, deep vein thrombosis, and sepsis. Systematic review (20), which included urological and genitourinary patients, revealed that intensive smoking cessation intervention with individual counseling and included pharmacotherapy, 4–8 weeks before surgery, reduced the risk of postoperative complications. Meta-analysis (21) of different surgical patients showed that quitting smoking 8 weeks before surgery has no negative impact on postoperative outcome. The guidelines for ERAS pathways in urology (6, 22) recommend smoking cessation 4–8 weeks before surgery.

Daily intake of more than two to three drinks decreases the immune response, prolongs bleeding time and increases endocrine stress response to surgery (23). More importantly, the preoperative 4-week-long abstinence from alcohol reduces exaggerated surgical stress response of alcohol abusers (24).

### Perioperative Nutritional Therapy

It has been shown (25) that the patients undergoing RC are at risk of malnutrition due to advanced age and prolonged hospital stay with high frequency of postoperative complications. In addition, these patients already have some degree of inflammation as a part of malnutrition. In a retrospective cohort study of 538 patients with RC (26), nutritional deficiency measured by preoperative weight loss, body mass index and also serum albumin, strongly predicts a 90-day survival, as well as poor overall survival.

Therefore, nutritional risk screening before a surgical procedure (27) is a highly recommended screening tool for establishing a possible risk of malnutrition. When it comes to establishing perioperative nutritional status, dietician referral 1 day before RC, represents yet another step forward in this whole process, just as it was already shown in the study of Arumainayagam et al. (28).

Perioperative nutritional therapy should be initiated before surgery if the patient is at nutritional risk or has malnutrition (29). The indication for preoperative nutritional therapy also exists if there is a trend of less than 50% of recommended oral food intake for more than 7 days, or food abstinence for more than 5 days.

In a randomized trial of Hamilton-Reeves et al., cystectomy patients showed different immune response to surgery and late infection (30) if perioperatively fed with specialized immunonutrition in intervention group. In accordance with the guidelines (29), perioperative or at least postoperative administration of specific formula enriched with immunonutritents should be given to malnourished patients undergoing major cancer surgery. However, the potential role, specific type, and financial aspects of preoperative nutritional therapy in RC patients are yet unknown.

### Bowel Preparation

The need for oral bowel preparation has been examined especially for ileum diversions of RC. Reduced bowel preparation in patients with mainly ileal conduit diversion of RC produced no detrimental effect on morbidity or mortality (28). In the study of Tabibi et al. (31), spillage was observed in all studied patients, in both bowel prepared ones and in those who had no preparation. The infection complications did not increase in the group without bowel preparation for cystectomy. In the randomized trial of Xu et al. (32), patients were randomized to a preoperative bowel preparation group and to another one with no preparation. It has been concluded that bowel preparation did not present any advantages in RC, neither with regard to patient recovery nor to complication occurrence.

The colorectal surgery meta-analysis (33) showed no clinical benefit from mechanical bowel preparation. This study also pointed out that inadequate bowel preparation with the presence of liquid content increases the risk of infections.

ERAS guidelines for RC (6) recommend that preoperative bowel preparation can be safely omitted.

### Thromboembolic Prophylaxis

Patients undergoing RC and RP are considered to be high risk of venous thromboembolism (VTE) because of the cancer disease and the surgery procedure lasting more than 120 min (34). Novotny et al. (35) revealed about 5% incidence of clinically significant deep vein thrombosis in RC patients, while according to Vukina et al. (36) incidence in open RP is 1–5% and just 0.5% in robotic RP. Despite importance of prevention of VTE in major urologic surgery, variations in utilization of prevention treatment are demonstrated and criticized (37).

European guidelines on perioperative VTE prophylaxis for fast-track surgery (34) recommend the first dose of LMWH 12 h before the procedure or 6–8 h after the procedure. In case of a planned neuraxial anesthesia, postoperative administration might be a preferred option. Furthermore, according to Sachdeva et al. (38), adding graduated compression stockings or compressive stockings enables more effective thromboprophylaxis.

ERAS Society guidelines for rectal/pelvic surgery (5) recommend taking into consideration extended prophylaxis for 4 weeks in patients with the increased risk of VTE. These recommendations are in accordance with the American College of Chest Physician guideline (39) and refer to high risk patients with cancer.

### Preoperative Fasting

It has been demonstrated (40) that long food and water abstinence produce stress and deteriorate surgical patient wellbeing. The European Society of Anaesthesiology fasting guidelines encourage patients to drink clear liquids (tea, coffee without milk and water) up to 2 h before the elective surgery. With the highest level of evidence, they recommend the prohibition of solid food 6 h before the elective operation. Patients with conditions such as gastro-esophageal reflux, obesity, diabetes, and pregnant women, who are not in labor, may have delayed gastric empting. More evidence is needed for fasting recommendations regarding these groups of patients.

In major urologic surgery, Rege et al. (41) reduced preoperative fasting period introducing clear liquids to 2 h before laparoscopic live kidney donor surgery in ERAS group of patients. They pointed out that the reduction of preoperative fasting period enhances patient’s comfort, reduces thirst and anxiety, thus facilitating faster recovery. In their study, patients with ERAS perioperative pathway had shorter length of hospital stay. On the other hand, in some early fast-track studies (42) of patients undergoing laparoscopic RP, liquid drinks were allowed only until midnight of the preoperative day. The only difference between the conventional and the ERAS group of patients in this study was that the ERAS group of patients was allowed to have lunch and soup for dinner, whereas the patients in the conventional group were not allowed to consume any food whatsoever after breakfast on the day before the surgery. Even without this preoperative element of ERAS pathway, the authors observed shorter length of hospital stay in the ERAS group.

According to previous discrepancies more evidence is needed about impact of shortening of fasting before major urologic surgery procedures.

### Preoperative Carbohydrate Loading

One of the main goals of ERAS protocols is the reduction of perioperative insulin resistance. Meta-analysis (43) of randomized controlled trials investigating preoperative oral carbohydrate treatment before elective surgery revealed significant reduction in the length of hospital stay of the patients receiving the treatment when compared with control groups. However, the authors pointed out that this was valid for patients undergoing major abdominal surgery, and they also emphasized that there was significant heterogenicity among different studies.

The extent of insulin resistance after surgery is proportional to the magnitude of the surgery (44) and blood loss (45). Both risk factors are present in RC (32) and other major urologic procedures. Several studies (40, 46, 47) of open RC conducted after the year 2010, used carbohydrate loading liquids 2 h before surgery with the aim of reducing postoperative insulin resistance. In the study of robotic-assisted laparoscopic cystectomy (48), 31 patients received carbohydrate loading at 6:00 p.m. the day before the surgery and at 5:00 a.m. on the day of the surgery. Patients within the study group showed significant differences in terms of mobilization within the room, the time to regular diet, and lower use of postoperative opioid analgesia. In other major urologic procedures, the reduction of insulin resistance was also found to be an important part of ERAS pathways. In the retrospective analysis (41) of patients undergoing laparoscopic live kidney donor surgery, preoperative carbohydrate loading liquids were used for ERAS pathway group. Further studies are needed to evaluate CHO loading for patients undergoing major urologic surgery.

### Antimicrobial Prophylaxis

European Association of Urology guidelines (49) suggested optional use of antimicrobial prophylaxis in RP and nephrectomy, since there are no studies on this issue. In RC patients, prophylaxis for both aerobic and anaerobic pathogens is recommended. The combination of cefuroxime and aminopenicillin/betalaktamase inhibitor plus metronidazole is recommended. In case of prolonged operation or important morbidity factors, antimicrobial prophylaxis might be prolonged to <72 h.

Prolongation of antibiotic prophylaxis is not recommended if urinary drainage is left in place after surgery.

### Prevention of Postoperative Nausea and Vomiting (PONV)

There is insufficient evidence regarding the incidence of PONV after urologic surgery. Shabsigh et al. (50) reported 29% of gastrointestinal complications from the overall number of complications, among which just 1.5% of patients were the ones with emesis. Nevertheless, PONV may intensify postoperative pain, wound dehiscence and hematoma and hence increase patient distress (51). With the aim of reducing the incidence of PONV, patient baseline risk should be assessed using validated score (52). Recommended risk scores are Apfel et al. (53) and Koivuranta et al. (54).

After establishing baseline risk, PONV is considered through all three parts of ERAS pathway, preoperative, intraoperative and postoperative. Prevention of PONV is accentuated especially in laparoscopic urologic surgery. In this way in the non-randomized retrospective analysis (43) of laparoscopic nephrectomy, antiemetics were started preoperatively with scopolamine patch, dexamethason and ondansetron were given intraoperatively and scopolamine and ondansetron postoperatively. For preoperative and postoperative phases, rescue antiemetics were also suggested. In conclusion, the rate of postoperative PONV was not analyzed albeit patients from intervention group had reduced length of hospitalization.

In the prospective randomized study (55) of RC patients, a guided intraoperative fluid therapy reduced the incidence of PONV (11 vs 3, *p* < 0.01 and 13 vs 1, *p* < 0.0001).

Yoo et al. (56) studied the incidence of PONV between group of patients with propofol total intravenous anesthesia (TIVA) and the group of patients with desflurane anesthesia for robot-assisted laparoscopic RP. The incidence of PONV was significantly lower in TIVA group both in post-anesthetic care unit and 1–6 h after the surgery.

## Intraoperative Period

This section reviews several important components of the intraoperative period (Table 1).

**Table 1:** Summary of intraoperative elements from published trials of enhanced recovery after surgery (ERAS) protocols for RC.

Authors	Arumainayagam et al.	Mukhtar et al.	Dutton et al.	Smith et al.	Daneshmand et al.	Persson et al.	Pang et al.
Reference/(*n*)	(28)/(112)	(46)/(77)	(12)/(165)	(57)/(133)	(58)/(110)	(13)/(70)	(14)/(453)
Non-ERAS *n*/ERAS *n*	56/56	26/51	0/165	69/37 ERAS 1, 27 ERAS 2	0/110	39/31	60/393
Surgery type	Open RC	Open RC	Open RC	Open RC	Open RC	Open RC	Open and robotic RC (425/28 ptc.)
Ileal condui/Neobl./Con.cu.div.	47/9/0	48/3/0	131/34/0	133/0/0	35/70/5	52/18/0	368/25/0
Antibiotic prophylaxis	Not specified	Not specified	Single dose cefuroxime, metronidazole	Not specified	+ Continued for 24 h	+ (100% ptc.)	Coamoxiclav24 h in men48 h in women
Analgesic method, *n* (%)							
Epidural	+	+	+ 25 ptc.	− ERAS 1− ERAS 2	–	+	–
RSC	–	–	+140 ptc.	+ ERAS 1+ ERAS 2	− Subfascial LA	−	+ (53% ptc.)
Other	−	−	−	−	iv. Acetaminophen	−	−
Avoiding of							
NGT	Not specified	+	+	+ ERAS 1+ ERAS 2	−	+	+ (84% ptc.)
Drains	−	+	−	−	−	+ (32% ptc.)	Consider omitting pelvic drain
Fluid/sodium management	Not specified	+	+ (GDFT)	+ ERAS 1, ERAS 2 (GDFT)	+ Monitored by SV and CVP	Not specified	+ (Limited fluid targeted to losses)
Intraoperative warming	Not specified	+	+	−	Not specified	+	+
Small incisions	Not specified	+	+	Change in surgical technique	Not specified	Not specified	+ (83% ptc.)
PONV prevention	Metoclopramide from day 1	+	Metoclopramide, omeprazole	Not specified	Not specified	+ (29% ptc.)	Antiemetics as needed
MgSO_4_ replacement	−	−	−	Infusion in ERAS 2	−	−	−

*RC, radical cystectomy; ptc., patients; Neobl., neobladder; Con.cu.div., continent cutaneous diversion; RSC, rectus sheath catheter; LA, local anesthetics; NGT, nasogastric tube; GDFT, goal directed fluid therapy; SV, stroke volume; CVP, central venous pressure; PONV, postoperative nausea and vomiting*.

### Perioperative Analgesia

The use of neuraxial anesthesia in RC and in RP patients is widely applied as one of the crucial elements of fast-track pathways (42, 59, 60). The American Pain Society (61) points out the importance of using neuraxial anesthesia in major thoracic and abdominal surgery, especially in patients with cardiologic and pulmonary morbidity or in those at risk of postoperative ileus (POI).

The epidural provides different positive effects on the general perioperative status of patients. Several studies reveal decrease in mortality (61, 62) as well as decrease in the risk of cardiovascular and respiratory (63) events in abdominal surgery. In RP and in RC patients, the epidural is related with reduced intraoperative blood loss (64, 65), earlier recovery of gastrointestinal peristalsis (66), and postoperative pain control (64). Still, Doiron et al. (67) observed no difference in length of stay, 30- and 90-day readmission rate, nor any influence on 30-day mortality among cystectomy patients with or without perioperative epidural. This is consistent with other studies (68, 69) in which the epidural was compared with intravenous patient-controlled analgesia.

It is extremely important to determine the aspects of using epidural in major urologic surgeries in the future. The level of epidural insertion for different urologic procedures is not precisely defined. For example, in some studies (60, 66) authors used Th9–11 for RC patients; however, in the study of Autran et al. (69) Th11-L2 was used. In RP studies, Shir et al. (64) used L3–L5, but Hong et al. (65) used Th12-L2. ERAS guidelines for RC (6) strongly recommend the use of thoracic epidural for 72 h, by extrapolating results from rectal surgery.

There are, however, other ways of administering perioperative analgesia in urology, which showed promising results. For example, in the study of Dutton et al. (12), out of 165 cystectomy patients that entered an enhanced recovery pathway, 140 patients had rectus sheath catheter (RSC) analgesia (Table 1). The authors switched from regional anesthesia to RSC blocks because of their numerous benefits. The advantages of RSC blocks have previously been studied (70, 71), and they include highly successful placement, patient safety, the possibility to use in patients taking antiplatelet medications, as well as the reliability during postoperative care.

### Intraoperative Fluid Therapy

The optimization of fluid therapy, as a part of fast-track pathways, aims at “zero balance,” maintaining preoperative fluid composition and weight (72). According to Gupta and Gan (73), maintenance fluid therapy in adult patients during major abdominal surgery should be accomplished with 1–3 ml/kg/h.

Intraoperative fluid therapy in RC was studied in the prospective study of Pillai et al. (55). Patients were randomized to receive standard intraoperative or esophageal Doppler guided fluid therapy. The study demonstrated improvements in gastrointestinal function with significant reduction of ileus, PONV and also wound infection in the intervention group. The trial patients received significantly greater volumes of intravenous fluid during the first operative hour. Authors postulated that this was the underlying reason for avoidance of occult splanchnic hypoperfusion and lowering of postoperative complications. It was also pointed out that timing of fluid administration may be the goal for tissue perfusion, rather than volume. The major limitations of this study were small number of patients and inclusion just of ASA1 and ASA2 patients.

In another randomized trial (74), patients were allocated to receive low volume of fluid therapy (1–3 ml/kg/h) with preemptive norepinephrine of 6 µg/kg/h intraoperatively. The intervention group had reduced complication rate and hospitalization time. Furthermore, in another study the authors (75) showed zero fluid balance and zero weight gain on the first postoperative day with the same intraoperative intervention.

In multivariate logistic regressions of Bazargani et al. (76), there was no significant link between higher intraoperative fluid intake and complications on the 30th and 90th days in 180 patients who underwent RC. It should be emphasized that this was ERAS pathway study for RC, *vice versa* to previously mentioned studies (55, 74). It was suggested that various measures of ERAS protocol attenuated possible negative effects of high fluid administration volumes. The controversy of fluid management in RC continues. Fluid restriction with possible silent or evident splanchnic ischemia and hypotension must be compared with fluid overload and interstitial and gut edema. Possible better monitoring of bowel perfusion and standardized protocols of intraoperative fluid administration in ERAS pathways in RC and other major urologic procedures is needed.

### Preventing Intraoperative Hypothermia

The maintenance of normothermia during surgery prevents high oxygen consumption, wound infection, bleeding, and pain. China’s national cross-sectional study (77) on 3,132 patients under general anesthesia showed increased ICU admissions and prolonged hospital stay in patients with intraoperative hypothermia.

The active warming strategies, such as the use of warm fluids and forced air warming (78), were more effective than passive warming in maintaining stable intraoperative hemodynamics and core temperature.

### Surgery Type

Compared with open surgery (79), minimally invasive surgery enhances patients’ recovery, due to less stress, and it also significantly lowers opioid requirements (80). According to systematic reviews (81, 82), the implementation of robotic RC has reduced blood loss and transfusion, inpatient narcotic requirements, time to regular diet, and length of hospital stay. In the prospective randomized study of Nix et al. (80), there was lower estimated blood loss and there were fewer narcotic requirements during robotic RC. Postoperative return of bowel function was more rapid in patients who underwent robotic RC. The authors believe that the probable reasons for the previously stated findings were lower degree of bowel manipulation, less fluid imbalance and lower overall opioid consumption. However, in addition to the benefits, it should also be stated that, according to some studies (79, 80), robotic cystectomy lasts significantly more and has similar rate of postoperative complications when compared with open RC.

Robot-assisted RP has gained popularity due to its benefits which are similar to those of robotic RC (56). Nevertheless, studies mainly investigate disease control and functional sequel. Maurice et al. (83) listed increased travel burden and limiting access to surgical care as disadvantages of RP. Further studies on the implementation of fast-track pathways in robotic prostatectomy are needed.

Studies of laparoscopic RC state different benefits. In the study (84) of 47 patients undergoing laparoscopic RC, before and after the implementation of the ERAS protocol, ERAS group had lower frequency of central vein catheter infection and paralytic ileus. Guan et al. (85) showed that patients with fast-track laparoscopic RC had shorter time to first flatus and regular diet, lower serum C-reactive protein and white blood count on the fifth and seventh day after surgery, as well as lower frequency of complications.

## Postoperative Period

Several elements related to postoperative period are reviewed here.

### Nasogastric Tube (NGT)

Early NGT removal was introduced in the study of Pruthi et al. (86) as a fast-track element for RC. The NGT was removed on the first day, and clear liquid was introduced on the second postoperative day. The main improvement was seen in postoperative morbidity. Park et al. (87) found out that early NGT removal after RC is not correlated with POI. In the prospective study (88), the authors examined the combination of metoclopramide and early nasogastric suction removal in RC patients and revealed reduction of postoperative atelectasis and earlier tolerance of solid food without complications, regarding bowel anastomosis. Retrospective analyses of RC patients (12) in the enhanced recovery program introduce “no routine NGT” as one step forward. The authors reserved the use of nasogastric suction only for patients with a POI. Outcomes of this study revealed no adverse effects on readmission and complications. It can be concluded that further studies will have objective to determine if the routine use of NGT in RC is necessary.

### Postoperative Analgesia

Epidural analgesia given during the period of 2–3 days after surgery, preferably without opioids, provides more efficient analgesia, compared with patient-controlled analgesia (89) in colorectal surgery. As far as urologic surgery is concerned, according to the study of Hong et al. (65), postoperative pain scores were lower in patients with combined general and epidural anesthesia, compared with RP patients with general anesthesia only. The authors concluded that this may be important for the reduction of the incidence of postoperative chronic pelvic pain. In the prospective, randomized double-blinded study (90), it was shown that continuous epidural infusion of local anesthetics and sufentanil alone or combined produced adequate analgesia for RP and nephrectomy. The authors found out that ropivacaine, combined with sufentanil, was the most preferable combination because of low incidence of motor block.

As it was mentioned before, RSC analgesia (12) for RC and transverses abdominis plane block (91) in RP are getting attention as alternatives to neuraxial anesthesia for perioperative analgesia. The idea of combining motor blocks with oral paracetamol/non-steroidal anti-inflammatory drugs (91) may potentially eliminate opioids from postoperative analgesia.

Optimal postoperative analgesia for major urologic surgery includes different techniques and different drugs. The introduction of new minimally invasive surgical techniques implies the use of different modalities; therefore, the specific role of certain combinations of analgesia regimens needs to be investigated in future.

### Prevention of POI

Postoperative ileus is a frequent gastrointestinal complication especially after RC. The incidence of POI in RC has been between 4 and 31% (13, 35). With the aim to define early postoperative morbidity after RC, Shabsig et al. (50) proposed the definition of ileus as “Inability to tolerate solid food by postoperative day five, the need to place NGT or the need to stop oral intake due to abdominal distension, nausea or emesis.” Proposed mechanisms for POI after RC (92) are fluid overload, electrolyte shifts, bowel manipulation, and opioid use. It has been theorized that the presence of urine in the operative field during RC delays resumption of the bowel motility (47).

It has been shown (13) that patients guided with ERAS pathway have significant reduction in the average time of the first passage of stool compared with pre-ERAS group. The prevention of POI involves a sum of benefits of ERAS elements. These are epidural perioperative analgesia (66, 92), optimization of intraoperative fluid therapy (55), minimally invasive approach to surgery, early NGT removal with early oral intake (13), and early mobilization.

Other measures used with the aim of promoting bowel function and ileus prevention are chewing gum and using alvimopan. In the study of robotic RC (93), patients that chewed gum had shorter time to first flatus in comparison with the standard ones.

In a retrospective study of Hamilton et al. (94), the alvimopan group of patients undergoing RC had significantly shorter average time of resuming a regular diet (5.3 vs 4.1 days, *p* < 0.01).

Finally, regardless of the great importance of POI regarding postoperative morbidity in ERAS for RC, its significance in other major urologic surgeries is still to be evaluated.

### Early Oral Intake and Postoperative Nutrition

The safety of early oral intake after bowel anastomosis was shown in several studies (95, 96). The guidelines for perioperative care in elective rectal/pelvic surgery recommend oral diet “*ad libitum*” 4 h after rectal surgery (5). Pruthi et al. (86) showed improvement in perioperative care in patients with RC by reducing time to clear liquid and regular diet. Early oral nutrition, as a part of multimodal approach (97), revealed reduced time to first flatus. Arumainayagam et al. (28) restarted clear fluids on the day of RC with other perioperative ERAS elements in 56 out of 112 patients. This study showed reduced total and postoperative hospital stay.

ESPEN guidelines for surgery (29) recommend that oral intake, including clear liquids, should be initiated within hours after surgery in most patients. ERAS society guidelines (6) for perioperative care after RC suggest that normal diet should be reestablished as soon as possible.

In the case of impaired oral or enteral tolerance for more than seven days, ESPEN guidelines (29) recommend adding parenteral nutrition. According to EPaNIC study (98) withholding of parenteral nutrition until day eighth appears to be superior strategy than early addition of parenteral nutrition. According to authors, early administration of parenteral nutrition suppresses autophagy thus preventing clearance of damaged cells and microorganisms. The study population was low severity critical care group of patients among which major surgical patients too. Moreover in the study of Roth et al. (99) in patients after RC with urinary diversion, immediate postoperative parenteral nutrition is associated with higher incidence of infection complications vs oral nutrition alone.

Early parenteral nutrition is beneficial in malnourished patients in whom oral or enteral nutrition is not feasible (29).

### Early Mobilization

Prolonged bed rest causes respiratory, musculoskeletal, and neuropsychological changes (100).

Primary conditions that have to be fulfilled before patient mobilization are the following ones: the gaining of patient’s motivation, postoperative pain relief, and the prevention of orthostatic intolerance (17). Furthermore, in the randomized study of Gatt et al. (101) on colonic surgery, the implementation of structured mobility plan with an active intervention of a physiotherapist resulted in longer period of time out of bed and in increased grip strength.

In the study of Pang et al. (14), the implementation of early mobilization of patients after RC along with other perioperative elements of the ERAS protocol reduces the length of hospital stay and the frequency of readmission. In this prospective study, on the first postoperative day, patients stayed out of bed for 6 h and walked 10–20 m, while on the second postoperative day they walked 100 m. Other authors (12, 13) also enlist early mobilization of patients into local ERAS protocol for cystectomy with similar results.

Early mobilization is emphasized in perioperative care of patients after RP as well. In the prospective randomized study of Gralla et al. (42), patients walked around their room and around the ward on the very day of the surgery. Patients from the conventional group were allowed only upright position on the same day.

Already defined as part of “proactive de-medicalization” (14), patient mobilization represents important prerequisite for stoma self-management and for the decrease in length of hospital stay.

### Urinary Drainage

Urinary bladder drainage is a routine procedure in major and urologic surgery. Optimal duration of catheter drainage is 1 day after colonic resections (102) and after pelvic surgery in patients with low risk of urinary retention (5, 6). Catheter removal on the first postoperative day after thoracic and abdominal surgery reduces the incidence of urinary tract infections.

The time period of urinary drainage in radical RC patients is vaguely defined in scientific literature. According to the study of Mattei et al. (102), the stenting of ureteroileal anastomosis resulted in decreased postoperative upper urinary tract dilatation; it improved postoperative bowel function and also decreased metabolic acidosis. The consensus statement about the exact timing of the stent removal in ileal conduit patients varies from 5 to 14 days. The urinary catheter in orthotopic neobladder is left for, at least, 14 days (103) after surgery.

Bearing in mind insufficient evidence (6) analyzed so far, this particular field of ERAS protocol needs to be studied in the future.

## Conclusion

Multimodal perioperative approach involves many evidence-based interventions with the aim of helping without doing any harm. Major urologic procedures, especially RC, represent a special challenge for future investigation in the ERAS era. Important fields for future investigation, regarding preoperative phase of surgery, are the following ones: the importance of nutritional therapy with the emphases on immune formulas, the omitting of preoperative bowel preparation, and the impact it will have on postoperative outcome, possible advantage of prolonged thromboprophylaxis regarding decreasing risk of VTE and further consideration of lowering insulin resistance. Intraoperative period studies will have to distinguish between the patients with possible risks of fluid overload and the need for guided fluid therapy and the patients who need special surgical techniques. New modalities of opioid sparing postoperative analgesia, the importance of implementing new drugs and special ERAS elements with the aim of preventing POI and the defining of optimal duration of urinary drainage, will be interesting issues to be studied in future, related with postoperative period in major urology. The main prerequisite for everything stated above is the increase of ERAS implementation in major urologic surgery.

## Author Contributions

NV conceived, designed, and wrote the manuscript. LD helped in analyzing the data.

## Conflict of Interest Statement

The authors declare that the research was conducted in the absence of any commercial or financial relationships that could be construed as a potential conflict of interest.
